# Review of "Image processing and analysis: variational, PDE, Wavelet, and Stochastic methods" by Tony F. Chan and Jianhong (Jackie) Shen

**DOI:** 10.1186/1475-925X-5-38

**Published:** 2006-06-09

**Authors:** Ming Jiang

**Affiliations:** 1 LMAM, School of Mathematical Sciences, Peking University, Beijing 100871, China

Image processing is an important component of modern technologies because human depends so much on the visual information than other creatures. Image is better than any other information form for us to perceive. Among our information about the world, 99% is perceived with our eyes [1]. Image processing has traditionally been an area in the engineering community. The basic tools are Fourier analysis with a long history and wavelet analysis which has become popular in the engineering community since 1980's. In the past few decades several advanced mathematical approaches have been introduced into this field, namely, the variational calculus, partial differential equations (PDE) and stochastic approaches (or statistical methods), and they have become important tools for theoretical image processing. From the cover, "*This book bridges contemporary mathematics with state-of-the-art methodologies in modern image processing while organizing the vast contemporary literature into a coherent and logical structure*."

In this book the authors present an integrated theory for image processing through the aforementioned approaches. From the preface, "*These diversified approaches are apparently distinct but in fact intrinsically connected. Each method excels from certain interesting angle or levels of approximations but is also inevitably subject to its limitations and applicabilities. On the other hand, at some deeper levels, they share common grounds and roots, from which more efficient hybrid tools or methods can be developed. This highlights the necessity of integrating this diversity of approaches."*

There is an introductory preface and 7 chapters, many illustration figures, an index and an extended bibliography of 330 entries.

The 1^st ^chapter, "*Introduction*", begins with an introduction of imaging science and image processing problems covered in the book: *image enhancement*, *image denoising*, *image deblurring*, *image inpainting *and *image segmentation*. It presents a methodological overview of the Fourier analysis, mathematical morphology, wavelet theory, stochastic approach, variational method, and partial differential equations. The authors conclude that those various approaches are intrinsically interconnected. There is one section about the organization of the book and one on the usage of the book for readers of different backgrounds.

The 2^nd ^chapter, "*Some Modern Image Analysis Tools*", reviews mathematical theories for the geometry of curves/surfaces, functions of bounded variations (BV), and Bayesian statistical inference, linear/nonlinear techniques of filtering/diffusion, and wavelets/multiresolution analysis. This chapter can also serve as a reference source for readers from other disciplines other than mathematics.

The 3^rd ^chapter, "*Image Modeling and Representation*", discusses five mathematical representations of images: deterministic models as functions of various smoothness, stochastic models with random fields, multiscale models based on the wavelet theory, level-set models by PDEs and Mumford-Shah models from the variational calculus. These representations link the mathematical theories in the 2^nd ^chapter with image processing problems in remaining chapters.

The 4^th ^chapter, "*Image Processing: Denoising*", first reviews the physical origin and stochastic models of image noise. It first recalls the classical denoising techniques such as linear low-pass filters and Wiener filters. The following modern denoising techniques are then presented: the seminal wavelet shrinkage technique, variational denoising methods based on TV and BV models, and algorithms from linear/nonlinear diffusion and scale space theory. There is an application to salt-and-pepper noise removal. The extension to multi-channel TV denoising is provided at the end of the chapter.

As in the 4^th ^chapter, the 5^th ^chapter, "*Image Processing: Deblurring*", also first reviews the physical background and stochastic models of image blur. The ill-posedness of the deblurring problem and regularization techniques are then briefly addressed, to which there are many books from different perspectives devoted. Image deblurring methods in this chapter include the classical Wiener filtration technique and variational methods for known PSFs. Blind deblurring for unknown PSFs with parametric and non-parametric PSFs are also presented.

The 6^th ^chapter, "*Image Processing: Inpainting*", is to investigate the image inpainting problem from the perspective of interpolation theory. "*The word inpainting is an artistic synonym for image interpolation*." [p.245] Various methods are studied including classical polynomial, trigonometric, spline and radial base function interpolations, and recent techniques with TV Radon measure, Munford-Shah-Euler model, wavelet, PDEs and Gibbs/Markov fields. Several applications of inpainting techniques are also discussed.

The 7^th ^chapter, "*Image Processing: Segmentation*", presents recent image segmentation methods including the active contour, Geman and Geman's Gibbs mixture model, Mumford-Shah's free boundary model, and multichannel logical segmentation technique. Unlike other chapters, this chapter does not provide a detailed background introduction on image segmentation. Readers can find relevant information in [1-3].

The book presents four powerful mathematical tools for image processing and analysis: *variational calculus*, *PDE*, *wavelet*, and *stochastic methods*. The authors have substantially contributed to the development of the theories and techniques. The book is well developed in depth, and to some extent self-contained. Generally speaking this book is in high quality and should be of general interests to the imaging processing community and relevant fields, though the underlying mathematics may be a burden to some readers. For example, some parts are quite mathematical, e.g., §7.1 on the monoids of occlusive preimages. The algorithms in the book may not be easy to implement because numerical techniques and programming tips are not easy for the complete novice even the authors have made a great and successful effort in the algorithm presentations. It would be very helpful if the authors can publish some demo codes on the Internet. Nevertheless the reviewer concurs with the following comment by Jean-Michel Morel in the cover, [this book is] "*A complete and very informative review of mathematical advances in image analysis in the past twenty years."*

From the cover, "*This book is written for graduate students and researchers in applied mathematics, computer science, electrical engineering, and other disciplines who are interested in problems in imaging and computer vision. It can be used as a reference by scientists with specific tasks in image processing, as well as by researchers with a general interest in finding out about the latest advances*." Interested readers are recommended to consult relevant books for image processing from the mathematical perspective [3-17].

## References

[B1] Russ JC (1995). The image processing handbook.

[B2] Marr D (1982). Vision : a computational investigation into the human representation and processing of visual information.

[B3] Morel JM, Solimini S (1995). Variational methods in image segmentation: with seven image processing experiments. Progress in nonlinear differential equations and their applications ; v 14.

[B4] Aubert G, Kornprobst P (2001). Mathematical problems in image processing: partial differential equations and the calculus of variations.

[B5] Dal Maso G, Tomarelli F (2002). Variational methods for discontinuous structures: international workshop at Villa Erba (Cernobbio), Italy, July 2001. Progress in nonlinear differential equations and their applications ; v 51.

[B6] Haar Romeny B (1997). Scale-space theory for computer vision: 1st International Conference, Scale-Space '97, Utrecht, The Netherlands, July 2-4, 1997: proceedings. Lecture notes in computer science ; 1252.

[B7] Nielsen M (1999). Scale-space theories in computer vision: 2nd international conference, Scale-Space'99, Corfu, Greece, September 26-27, 1999: proceedings. Lecture notes in computer science, 1682.

[B8] Kerckhove M (2001). Scale-space and morphology in computer vision: 3rd International Conference, Scale-Space 2001, Vancouver, Canada, July 7-8, 2001: proceedings. Lecture notes in computer science ; 2106.

[B9] Griffin LD, Lillholm M (2003). Scale space methods in computer vision: 4th international conference, Scale Space 2003, Isle of Skye, UK, June 10-12, 2003: proceedings. Lecture notes in computer science, 2695.

[B10] Kimmel R, Sochen N, Weickert J (2005). Scale space and PDE methods in computer vision: 5th international conference, Scale-Space 2005, Hofgeismar, Germany, April 7-9, 2005: proceedings. Lecture notes in computer science, 3459.

[B11] Osher S, Paragios N (2003). Geometric level set methods in imaging, vision, and graphics.

[B12] Paragios N, Chen Y, Faugeras O (2006). Handbook of mathematical models in computer vision.

[B13] Ronse C, Najman L, Decencière E (2005). Mathematical morphology: 40 years on : proceedings of the 7th International Symposium on Mathematical Morphology, April 18-20, 2005. Computational imaging and vision ; v 30.

[B14] Sporring J (1997). Gaussian scale-space theory. Computational imaging and vision ; v 8.

[B15] Weickert J (1998). Anisotropic diffussion in image processing. ECMI Series.

[B16] Winkler G (2003). Image analysis, random fields and Markov chain Monte Carlo methods : a mathematical introduction. Applications of mathematics, 27.

[B17] Paragios N (2005). Variational, geometric, and level set methods in computer vision: 3rd international workshop, VLSM 2005, Beijing, China, October 16, 2005: proceedings. Lecture notes in computer science, 3752.

